# Immune Suppressive Effects of Plasma-Derived Exosome Populations in Head and Neck Cancer

**DOI:** 10.3390/cancers12071997

**Published:** 2020-07-21

**Authors:** Inga J. Beccard, Linda Hofmann, Jan C. Schroeder, Sonja Ludwig, Simon Laban, Cornelia Brunner, Ramin Lotfi, Thomas K. Hoffmann, Edwin K. Jackson, Patrick J. Schuler, Marie-Nicole Theodoraki

**Affiliations:** 1 Department of Otorhinolaryngology, Head and Neck Surgery, University of Ulm, 89070 Ulm, Germany; inga.beccard@uni-ulm.de (I.J.B.); linda.hofmann@uni-ulm.de (L.H.); jan.schroeder@uni-ulm.de (J.C.S.); simon.laban@uniklinik-ulm.de (S.L.); Cornelia.Brunner@uniklinik-ulm.de (C.B.); T.hoffmann@uniklinik-ulm.de (T.K.H.); patrick.schuler@uniklinik-ulm.de (P.J.S.); 2 Department of Otorhinolaryngology, Head and Neck Surgery, University Hospital Mannheim, 68167 Mannheim, Germany; sonja.ludwig@umm.de; 3 Institute for Clinical Transfusion Medicine and Immunogenetics Ulm, German Red Cross Blood Services Baden-Württemberg-Hessen, 89081 Ulm, Germany; r.lotfi@blutspende.de; 4 Institute for Transfusion Medicine, University Hospital Ulm, 89081 Ulm, Germany; 5 Department of Pharmacology and Chemical Biology, University of Pittsburgh School of Medicine, Pittsburgh, PA 15260, USA; edj@pitt.edu

**Keywords:** tumor-derived exosomes, immune regulation, HNSCC, liquid biopsy

## Abstract

Plasma-derived exosomes of head and neck squamous cell carcinoma (HNSCC) patients carry inhibitory factors mediating immune suppression. Separation of tumor-derived exosomes (TEX) and non-TEX may assist in a better understanding of their respective parental cells. Here, we evaluate the impact of TEX or hematopoietic-derived exosomes on immune suppression. We evaluated apoptosis in CD8+ T cells, conversion of CD4+ T cells to regulatory T cells (T_reg_), and adenosine production by TEX, non-TEX, or total exosomes. Exosome protein cargo was significantly higher in total and CD45(−) exosomes from high stage compared to low stage patients. Furthermore, total and CD45(−) exosomes of high stage patients induced more apoptosis in CD8+ T cells than their low stage counterparts. CD69 suppression, a marker of reduced CD8+ T cell activation, was only mediated by CD45(−) exosomes. All fractions induced T_reg_ differentiation, defined by CD39 expression, but only CD45(−) exosomes showed a stage-dependent conversion. CD45(−) exosomes produced higher adenosine concentrations than CD45(+) exosomes, concluding that adenosine production measured in total exosomes mainly derives from TEX. The presented results show significant induction of immune suppression by TEX in HNSCC. This immunosuppressive effect supports the potential role of exosomes as liquid biomarkers for disease stage and level of immune suppression.

## 1. Introduction

Head and neck squamous cell carcinomas (HNSCC) are highly aggressive and immunosuppressive malignancies [1,2,3]. Even though there is a broad variety of therapeutic approaches available, the outcome for advanced HNSCC is still very poor, with five-year survival rates being as low as 40% [4] due to early lymph node metastasis and locoregional recurrences [5]. 

One of the predictors for evasion of antitumor T cell response is an imbalance toward regulatory T cells (T_reg_) as opposed to CD8+ effector T cells in the tumor microenvironment (TME) [6,7]. T_reg_, characterized by the expression of ectonucleoside triphosphate diphosphohydrolase-1 (CD39) [8], not only play an important role in upholding this balance but also take part in the production of immune-suppressive factors such as adenosine, a molecule hydrolyzed from adenosine triphosphate (ATP) by CD39 and CD73 through an intermediate step via adenosine diphosphate (ADP) and 5′ adenosine monophosphate (AMP). It is degraded to inosine by adenosine deaminase (ADA), associated with membrane anchor protein CD26 [8]. The presence of adenosine in the TME of HNSCC has been linked to tumor progression and poor outcome [9]. Therefore, influencing factors on the T cell balance are of great importance to broaden our understanding of tumor-host interaction. Among other mechanisms contributing to immune suppression in the TME of HNSCC, extracellular vesicles (EVs) such as exosomes have been identified as carriers and producers of immunosuppressive molecules. Exosomes, as the smallest vesicles derived from the endocytic compartment, have a diameter between 30 and 150 nm and carry endocytic markers such as TSG 101 and tetraspanins [10,11]. Due to their unique origin, they also carry molecular and genetic content that is representative of their parent cells and present an important way of intercellular communication [12]. In plasma of HNSCC patients, significantly more exosomes have been found than in healthy donors [13], correlating with tumor stage and disease activity [14]. Through interaction with immune cells and non-immune cells in the TME, exosomes are able to reprogram and alter cellular functions [15,16,17]. This can lead to suppression of immune response and promotion of tumor growth [18]. With evolving methods for exosome isolation from plasma, both characterization of molecular content by Western blot, flow cytometry, or co-incubation assays [14,19] and separation into different subgroups have been achieved [20]. Plasma-derived exosomes originate from a multitude of different parent cells. Exosomes released from immune cells and tumor cells are of special interest due to the unique insights they provide into immune regulation and tumor status/progression in cancer patients. Previously the separation of CD3(+) T cell-derived exosomes and CD3(−) exosomes, enriched in TEX, helped to gain insights into exosomal signaling pathways of reprogrammed immune cells [20]. In patients with HNSCC, up to 50% of all exosomes were CD3(+) and, therefore, T cell-derived, whereas only 20-30% of exosomes isolated from healthy donors originate from T cells [20]. Both T cell-derived CD3(+) and non-T cell-derived CD3(−) exosomes were able to induce apoptosis and suppress CD69 expression in activated CD8+ T cells. However, the CD3(−) fraction induced higher levels of apoptosis in CD8+ T cells independent of immunosuppressive content [14,20]. Furthermore, upregulation of CD39 on CD4+ T cells with a consecutive increase of adenosine levels in the presence of exosomes from HNSCC cell lines and patients has been shown [20,21]. These results emphasize the importance of TEX-enriched fractions and immune cell-derived exosomes as influencing factors on immune response mechanisms in the setting of cancer. In contrast to other cancer types such as melanoma (CSPG4) or prostate cancer (PSA), there is no specific tumor marker available for the identification of HNSCC. Hence, separation of TEX-only fractions presents a difficult task. To further characterize the effect of a higher TEX-enriched fraction assuming a higher tumor load, we used a previously described immune capture approach. As CD45 is ubiquitously expressed on all nucleated hematopoietic cells [22] including B and T cells but not on squamous cells, we used this antigen for immune capture and enrichment of TEX. In this study, we analyze the effect of CD45(−) TEX-enriched fraction on various T cell populations to understand further the TME-modulating effects achieved by exosomes. Furthermore, we evaluate the potential of plasma-derived exosome fractions as easily accessible liquid biomarkers for disease stage and immune suppression. 

## 2. Results

### 2.1. Clinicopathological Characteristics of HNSCC Patients 

The clinicopathological data for 41 patients included in this study are listed in Table 1. Age ranged from 43 to 85 years, with 71% of all patients being younger than 65 years. Patients were predominantly male, and tumors were mainly located in the pharynx (52%), oral cavity (34%), and larynx (12%). All blood samples were collected prior to any therapy. Nineteen patients (46%) presented with Union for International Cancer Control (UICC) low stage and 22 patients (54%) with UICC high stage disease. 

### 2.2. Characteristics of Plasma-Derived Exosomes

**Figure 1:**
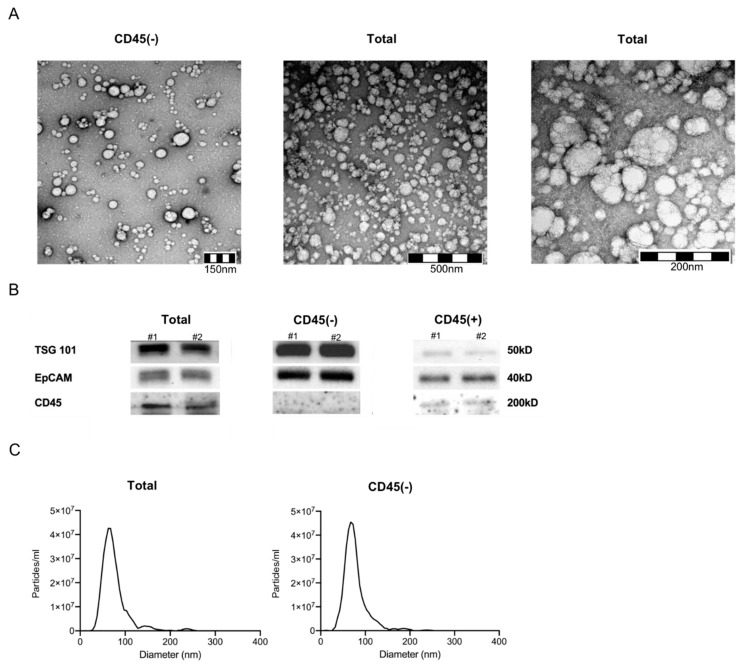
Exosome characterization. (**A**) Representative transmission electron microscopy pictures of CD45(−) (left) and total (middle and right) exosomes. (**B**) Western blot for detection of TSG 101, EpCAM and CD45 of total, CD45(−) and (+) exosomes from plasma of head and neck squamous cell carcinoma (HNSCC) patients (#1&2 high stage). Note that no CD45 signal was detected when using CD45(−) exosomes. Uncropped Western blots can be found at Figure S1. (**C**) Nanoparticle tracking analysis with ZetaView of total (left) and CD45(−) exosomes (right). Vesicles between 30 and 200 nm diameter were detected.

Exosomes isolated from plasma of HNSCC patients using mini size exclusion chromatography (mini-SEC) met the criteria attributed to exosomes as specified by the MISEV 2018 guidelines [23] (Figure 1). Transmission electron microscopy (TEM) confirmed vesicular morphology and vesicle diameters between 30 and 220 nm (Figure 1A). Western blots of exosomes confirmed the presence of TSG 101 as an endocytic marker (Figure 1B). Furthermore, CD45 was only present in CD45(+) and total exosome samples, corroborating successful separation of exosomes into CD45(+) and CD45(−) fractions (Figure 1B). Nanoparticle tracking analysis (NTA) confirmed a size range between 30 and 200 nm (Figure 1C). 

### 2.3. Association of Exosomal Protein Levels with Clinicopathological Parameters

**Figure 2:**
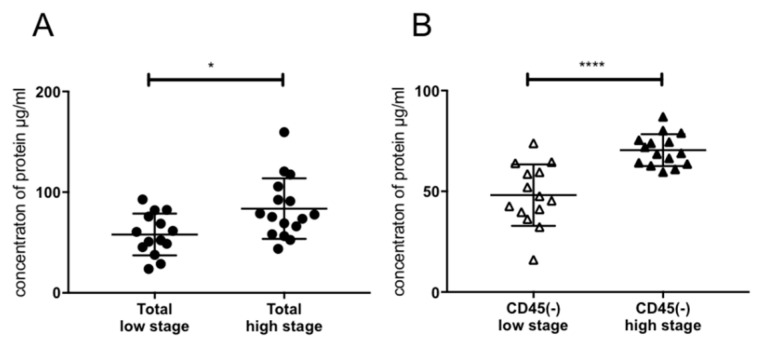
Exosomal protein concentration. (**A**) Protein concentration of total exosomes from plasma of high and low stage HNSCC patients, *n* = 30 (**B**) CD45(−) exosome fraction of high and low stage patients, *n* = 29. *p* values were determined with Mann-Whitney test, with * *p* < 0,05, **** *p* < 0,0001. Bars represent mean with standard deviation (SD).

Protein levels in exosome samples as a marker of tumor load were examined for association with tumor stage. Figure 2A shows a significantly higher protein content in exosomes of UICC high stage (III/IV) patients as opposed to UICC low stage (I/II) patients as demonstrated before [13]. The CD45(−) TEX-enriched fractions showed a higher exosome load in high stage compared to low stage patients, corresponding to a higher tumor load (Figure 2B).

### 2.4. Induction of Apoptosis in Activated CD8+ T Cells by Plasma-Derived Exosomes

**Figure 3:**
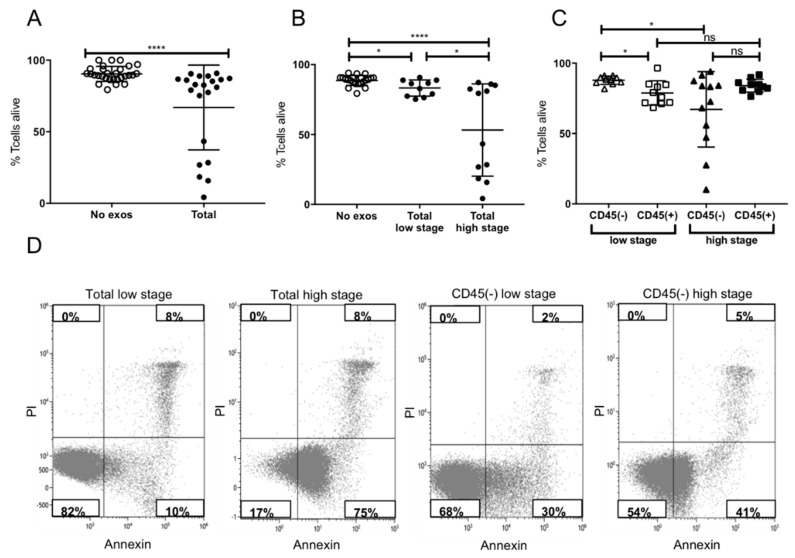
Induction of apoptosis in activated CD8+ T cells. (**A**) Induction of apoptosis after incubation with total exosome fractions. (**B**) Induction of apoptosis after incubation with total exosomes of low stage vs. high stage patients. (**C**) Levels of apoptosis after co-incubation with CD45(−) or CD45(+) exosomes of low or high stage patients. Note that only CD45(−) exosomes induced stage-dependent apoptosis, whereas CD45(+) exosomes showed no significant difference between stages. CD45 (+) exosomes of low stage patients induced more apoptosis than CD45(−) exosomes, whereas no significant difference was visible between those of high stage patients; *n* = 29. *p* values were determined with Mann-Whitney test, with * *p* < 0.05, **** *p* < 0.0001. Bars represent mean with standard deviation (SD). (**D**) Representative flow cytometry plots depicting CD8+ T cells stained with Annexin/Propidium Iodide (PI) after incubation with total and CD45(−) exosomes of Union for International Cancer Control (UICC) low or high stage patients.

Induction of apoptosis in activated CD8+ T cells by plasma-derived exosomes of high and low stage patients was tested. Incubation with total exosomes of HNSCC patients induced significant levels of apoptosis compared to no-exosome controls, which was confirmed in this study (Figure 3A). When incubating activated CD8+ T cells with exosomes of UICC high and low stage patients, significant differences in apoptosis were visible. Total exosomes of high stage patients induced significantly more apoptosis in T cells compared to total exosomes of low stage patients (Figure 3B). This stage-dependent apoptosis was reflected in the CD45(−) exosome fractions. In contrast, CD45(+) exosomes showed a stage independent induction of apoptosis (Figure 3C). Comparing the induction of apoptosis by CD45(−) and CD45(+) exosomes of high stage patients, no significant difference was visible with both fractions inducing similar levels of apoptosis. In contrast, the CD45(+) fraction of low stage patients induced higher levels of apoptosis compared to the TEX-enriched fraction (Figure 3C). Figure 3D presents exemplary flow cytometry plots, visualizing the difference between apoptosis induction by total exosomes of UICC low and high stage patients. 

### 2.5. Suppression of CD8+ T Cell Activation by Plasma-Derived Exosomes

**Figure 4:**
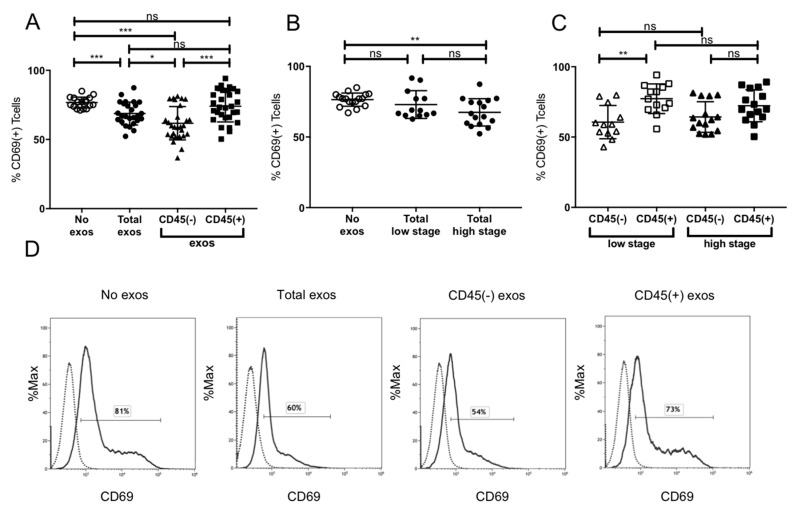
CD69 expression by activated CD8+ T cells. (**A**) CD69 expression after incubation with total, CD45(−) and CD45(+) exosome fraction of patients of all stages (**B**) CD69 expression after incubation with total exosomes of low stage vs. high stage patients. (**C**) Incubation of CD8+ T cells with CD45(−) or CD45(+) exosomes of low or high stage patients.; *n* = 29. *p* values were determined with Mann-Whitney test, with * *p* < 0.05, ** *p* < 0.005, *** *p* < 0.0005. Bars represent mean with standard deviation (SD). (**D**) Representative flow cytometry histograms depicting CD69 levels on CD8+ T cells after incubation with phosphate-buffered saline (PBS), total, CD45(−), and CD45(+) exosomes (left to right) are shown. The solid line represents the CD69 signal, the dashed line represents the unstained control. The respective CD69 positive signal is displayed in percent.

The induction of CD69 expression on cytotoxic CD8+ T cells as a marker of activation was evaluated after incubation with exosomes. Incubation of CD8+ T cells with total exosomes of HNSCC patients showed significant inhibition of CD69 expression (Figure 4A). Total exosomes showed significant inhibition of CD69 expression. However, the CD45(−) exosome fraction induced an even higher suppression of CD69 in CD8+ T cells in contrast to CD45(+) exosomes, which did not affect T cell activation levels at all (Figure 4A). Only exosomes of high stage patients induced a significant reduction of CD69 expression (Figure 4B). Looking at the subgroups no inhibitory influence of CD45(+) exosomes on CD69 levels and, therefore, on CD8+ T cell activity was evident. Interestingly, CD45(−) fractions of both low and high stage patients induced similar levels of CD69 inhibition (Figure 4C). Especially CD45(+) and CD45(−) exosomes of low stage patients presented significantly different levels of CD69 suppression (Figure 4C). Exemplary flow cytometry histograms are shown in Figure 4D. 

### 2.6. Induction of CD39+ Treg Differentiation 

**Figure 5:**
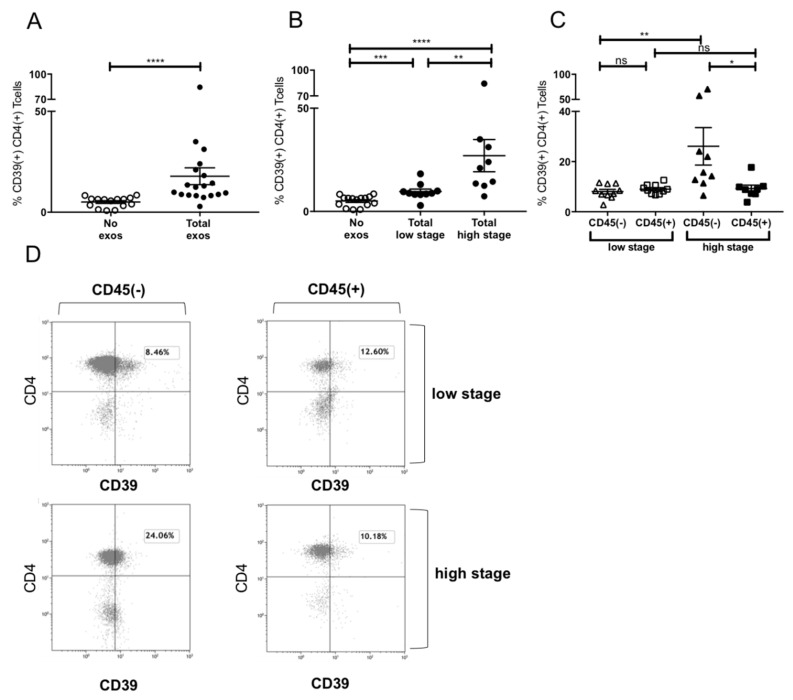
Induction of CD39+ T_reg_ differentiation by plasma-derived exosomes. (**A**) Expression of CD39 after incubation with total exosome fraction of patients of all stages (**B**) Expression of CD39 after incubation with total exosomes of low stage vs. high stage patients. (**C**) Expression of CD39 after incubation with CD45(−) and CD45(+) exosomes of low stage and high stage patients. Note that only incubation with CD45(−) exosomes showed significant stage-dependent differences.; *n* = 18. *p* values were determined with Mann-Whitney test, with * *p* < 0.05, ** *p* < 0.005, **** *p* < 0.0001. Bars represent standard error of mean (SEM). (**D**) Representative flow cytometry plots depicting CD39 expression of CD4+ T cells after incubation with CD45(−) (left) or CD45(+) (right) exosomes of UICC low stage (top) or high stage (bottom) patients.

It was previously reported that exosomes from HNSCC upregulated CD39 expression dependent on their immunoinhibitory molecular content [20]. We confirmed CD39 induction on CD4+ T cells by total exosomes (Figure 5A). Furthermore, a stage-dependent induction of CD39 was visible (Figure 5B), with total exosomes of UICC high stage patients inducing significantly more CD39 than those of low stage patients (Figure 5B). When evaluating the CD39 induction by exosome subpopulations, the CD45(+) fraction induced stage-independent low levels of CD39 expression, whereas the CD45(−) high stage fraction induced a relevant increase in CD39 (Figure 5C). Exemplary flow cytometry plots are visible in Figure 5D visualizing the difference between CD39 induction on CD4+ T cells by CD45(+) and CD45(−) exosomes of UICC low and high stage patients.

### 2.7. Production of Adenosine by Plasma-Derived Exosome Populations

**Figure 6:**
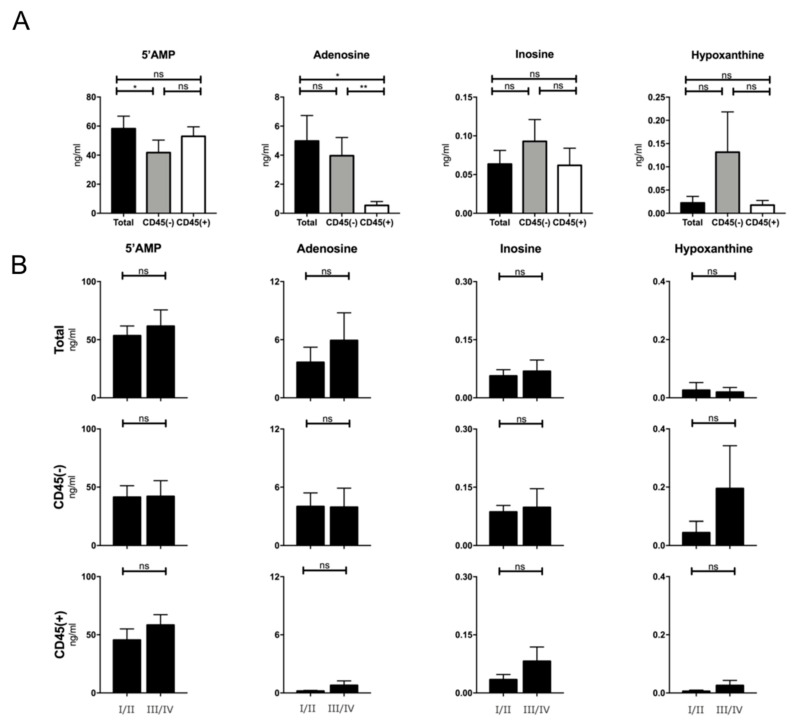
Production of nucleosides by exosomes of all stages. (**A**) Respectively, 5′ adenosine monophosphate (5′AMP), Adenosine, Inosine, and Hypoxanthine production by total, CD45(+) and (−) exosome fractions. The different exosome fractions showed significant differences between 5′AMP and adenosine production; *n* = 18; (**B**) Production of nucleosides in ng/mL by total exosomes comparing UICC low and high stage patients; *n* = 19. Mann-Whitney with * *p* < 0.05, ** *p* < 0.005, bars represent standard error of mean (SEM).

It has been shown previously that exosomes are independent producers of adenosine and other nucleosides due to the presence of CD39 and CD73 on their surface [8,13,20]. Here, we evaluated the nucleoside production by exosomes of different fractions and tumor stage. Figure 6A shows a tumor-stage independent nucleoside production of total exosomes. No significant difference in production of inosine and hypoxanthine was visible. CD45(−) exosomes showed significantly lower 5′AMP levels than CD45(+) and total exosomes. Total and CD45(−) exosomes produced higher adenosine levels compared to CD45(+) exosomes, concluding that the majority of adenosine production derives from the CD45(−), TEX-enriched, fraction. No significant differences were visible when stratifying patients according to their tumor stage (Figure 6B).

## 3. Discussion

As exosomes isolated from plasma are a mix of extracellular vesicles originating from numerous different cells [24], the assignment of effects to a certain cellular subpopulation using total exosome fractions is not possible. However, exosomes are likely to express markers of their parent cell, aiding in the selection of specific subpopulations. With bead-based immunoaffinity selections the separation of various subpopulations is possible, including CD3(+) T cell-derived, CD45(+) hematopoietic-derived and CD45(−), or CD44v3(+) TEX-enriched fractions [20,25]. Here, we used our previously developed immune capture approach based on CD45 separation to enrich TEX and analyze their immune-modulatory effects compared to the CD45(+) fraction. 

We were able to confirm previous findings showing elevated protein levels in exosome samples of HNSCC patients and a correlation with tumor stage [13], but not only for total exosome samples but also for CD45(−) exosomes (Figure 2). This corroborates the hypothesis that CD45(−) exosomes are highly enriched in TEX, quantitatively representing the tumor stage of the patient and suggesting possible clinical applications as a diagnostic biomarker.

Since a strong presence of CD8+ T cells in the TME is generally associated with a better prognosis [26,27] and intra-tumoral accumulation of T_reg_ predicts resistance to treatments and accelerated cancer progression [28], evaluation of regulating mechanisms of immune cell populations, especially T cell fractions, remains important. 

We and others have shown previously that total exosomes of HNSCC patients induce apoptosis in activated CD8+ T cells [13,20]. Here, we showed a stage-dependent induction of apoptosis by total exosome fraction and further showed a stage-dependent induction of apoptosis by CD45(−), TEX-enriched exosomes (Figure 3). CD45(+) exosomes also induced apoptosis, which is in line with previously published characteristics of CD3(+), T cell-derived exosomes [20]. Exosome-induced apoptosis originated mainly from the CD45(+) fraction in low stage patients, whereas both, CD45(+) as well as CD45(−) exosomes of high stage patients, induced similar levels of apoptosis. This might be due to a higher, stage-dependent variability of immune-suppressive molecular cargo in CD45(−) TEX-enriched exosomes as opposed to a more static molecular cargo of CD45(+) exosomes. These findings are in line with the results of Ludwig et al. [13] showing increased Fas and FasL expression on UICC high stage plasma-derived exosomes with increased induction of caspase 3/7 activity in CD8+ T cells. The supposed differences between induction of apoptosis by CD45(+) exosomes of low stage patients (Figure 3C) and the CD8+ T cell by CD69 induction (Figure 4C) might be due to simultaneous expression of FasL and expression of CD3, as CD3 is a known inducer of CD69 expression upon T cell receptor (TCR) engagement [29].

Besides tumor stage-dependent induction of apoptosis by CD45(−) exosomes, they were also responsible for suppression of CD8+ T cell activation as reflected by a high suppression of CD69 on activated CD8+ T cells (Figure 4). Confirming previous results, there was no CD69 suppression detected by total exosomes of UICC low stage patients [14]. However, we found that the CD45(−) exosome fraction suppressed T cell activation independently of tumor stage, indicating that CD69 suppression is a more stationary effect, which is induced by all TEX. It is possible that CD45(+) exosomes, especially those of UICC low stage patients, carry immune stimulatory molecules, which compensate for the effects of CD45(−) exosomes [20]. We have previously shown that CD3(+) exosomes from UICC low stage patients carried higher levels of immune-stimulatory molecules OX40 and lower levels of immune-suppressive factors as cyclooxygenase-2 (COX-2) [20]. Similar results have been reported in melanoma patients, with non-tumor-derived exosomes carrying a higher load of immune-stimulatory molecules than tumor-derived exosomes [30]. 

In contrast to CD8+ T cells, which play an immune-supportive role in the TME, adenosine-producing CD39+ and CD73+ T_reg_ are important factors in the inhibition of anti-tumor immunity and associated with poor outcome [8,9].

Exosomes were shown to be independent producers of adenosine by surface expression of CD73 and CD39 and to induce differentiation of CD4+ T cells to CD39+ T_reg_ [8,20,31], potentiating immune suppression in the TME. Here, we showed that induction of CD39 on CD4+ T cells by total plasma-derived exosomes was dependent on the tumor stage at which exosomes were isolated (Figure 5). Even more, T_reg_ differentiation was mainly caused by CD45(−) TEX-enriched exosomes. CD45(+) exosomes did not show any effect on T_reg_ differentiation independently of UICC stage. Especially exosomes carrying highly immunosuppressive cargo were reported to induce CD39 expression on CD4+ T cells [20], with the assumption that the cargo of CD45(−) exosomes of high stage patients is especially enriched in immune-suppressive molecules, inhibiting an anti-tumor immune response in the TME.

Not only CD4+ T_reg_ but also exosomes themselves contribute to adenosine production in the TME [32], as they have been shown to carry the enzymatically active ectonucleotidases CD39 and CD73 and produce adenosine in the presence of exogenous ATP [8,31].

Significant differences in the production of adenosine were observed between total, CD45(+), and CD45(−) exosomes regardless of UICC stage. CD45(+) exosomes produced significantly less adenosine than CD45(−) and total exosomes, suggesting TEX as the main exosomal adenosine producers. Similar results were reported previously, showing a high adenosine production by CD3(−) TEX-enriched exosomes in contrast to almost no production by CD3(+) T cell-derived exosomes [31]. However, here we observed an even higher adenosine production by CD45(−) exosomes compared to CD3(−) fractions [31]. This may be due to a higher TEX-ratio in the first group by the exclusion of all hematopoietic cells compared to exclusion of only T cells in the CD3(−) fraction. 

Due to their unique origin and molecular profiles, exosomes have emerged as potential biomarkers in liquid biopsies [18,33]. Previously, not only the TEX-enriched fraction but also the immune cell-derived fraction had been investigated as liquid biopsy markers as well as therapy monitoring tools [13,31,34]. Our results and experience with the methodology concerning CD45(+) and CD45(−) exosomes suggest that these fractions can very well be used in a clinical setting to monitor therapy response during treatment as well as aid in staging procedures beforehand.

## 4. Materials and Methods 

### 4.1. Patients

Aliquots of 1mL plasma samples, stored at −80 °C, from 41 HNSCC patients seen between 2014 and 2019 at the Department of ENT, Head and Neck Surgery, University of Ulm were used for exosome isolation. The collection of blood samples and access to clinical data for research purposes was approved by the Ulm University ethics committee under proposal number 90/15. Additionally, blood of healthy controls was processed for T cell isolation. 

### 4.2. Exosome Isolation by Mini Size Exclusion Chromatography (Mini-SEC)

Exosomes were isolated using the mini-SEC method, as previously established, optimized, and described [13] (EV-TRACK ID: EV160007). Briefly, plasma samples were pre-cleared by centrifugation at 2000 *g* and 100,000 *g* followed by ultrafiltration using a 0.22 µm filter [13]. 1mL of pre-cleared plasma was placed on a mini-SEC column and eluted with phosphate-buffered saline (PBS). The 4th void volume fraction enriched in intact and functionally active exosomes was collected as previously described [13].

### 4.3. Characterization of Plasma-Derived Exosomes

The isolated exosomes were characterized according to MISEV 2018 standards [23] using (a) nanoparticle tracking (NTA), (b) transmission electron microscopy (TEM), (c) and Western blot analysis.

#### 4.3.1. Nanoparticle Tracking 

NTA was performed using the Zeta View PMX-220 (Particle Metrix, Inning, Bayern, Germany). Freshly isolated exosomes were diluted to a concentration of 1:100,000 in 1 mL PBS and loaded into the cell. Measurements were taken at 11 different positions throughout the cell. At each position, three cycles of records were taken. The instrument was operated at the following parameters: temperature of 25 °C, sensitivity of 85, and a shutter of 100. As a control, PBS and 100 nm polystyrene beads were used. The nanoparticle size ranges and concentrations were calculated using ZetaView 8.05.11 software.

#### 4.3.2. Transmission Electron Microscopy

Freshly isolated exosomes were layered on copper grids in a multi-step procedure and stained with 1% uranyl acetate in ddH_2_O. Immediate visualization by use of the transmission electron microscope followed. Pictures of vesicles, diameters ranging from 40 to 160 nm, were taken. 

#### 4.3.3. Western Blot

Exosomes were lysed in Lane Marker Reducing Sample Buffer (Pierce, Thermo Scientific), Waltham, MA, USA) separated on 12% SDS/PAGE gels (Bio-Rad, Hercules, CA, USA), always applying 10 µg protein per lane, and transferred onto a nitrocellulose membrane using the Transblot Turbo System (Bio-Rad). The membrane was incubated at 4 °C overnight with either TSG 101 antibody (1:500, PA31260, Thermo Scientific), CD45 (1:50, MA5-13197, ThermoFisher Scientific), or Epithelial cell adhesion molecule (EpCAM) (1:500, MA5-13917, ThermoFisher Scientific). After washing, a corresponding secondary antibody (1:10,000, 31450 and 31460, anti-rabbit or anti-mouse, ThermoFisher Scientific,) was added for 1 hour at room temperature (RT), and the membrane was developed with Super Signal West Dura Extended Duration Kit (Thermo Scientific) and analyzed using a ChemiDoc (BioRad).

### 4.4. BCA Protein Assay and Exosome Concentration

Pierce BCA protein assay kit (Pierce Biotechnology, Rockford, IL, USA) was used to determine the protein concentration of the isolated exosome fraction #4, following the manufacturer’s instructions. Concentration of exosomes was performed using centrifugal filters (cut-off 100kDa, Amicon Ultra by Merck Millipore). 

### 4.5. Separation of Total Exosomes Using Biotinylated CD45 Antibodies

Total exosomes were incubated with biotinylated CD45 Ab (clone H130, 304004 Bio Legend, San Diego, CA, USA) at dilution of 1:25 for 16h on a shaker at 4 °C. Pre-washed ExoCap Beads (Medical&Biological Laboratories Co. Ltd., Naka-ku, Nagoya, Aichi, Japan) were added to the sample and incubated another 2h on the shaker at RT. Separation into CD45(−) and CD45(+), bead-bound, exosome fractions was performed using a MagRack 6 magnetic stand (GE-Lifesciences, Chicago, IL, USA).

### 4.6. Functional Assays

#### 4.6.1. Apoptosis and CD69 Induction in CD8^+^ Cells by Patients’ Exosomes

CD8^+^ T cells (2 × 10^6^/mL), isolated with CD8+ T cell isolation kit (17953, Stemcell Technologies, Vancouver, Canada) according to manufacturer’s instructions, were activated using CD3/CD28 T-cell activator (25 μL/mL, Stemcell Technologies) and 0.7 µL IL2 (2 × 10^5^ U/ml, R&D Systems, Minneapolis, MN, USA) in RPMI supplemented with exosome-depleted FBS (Gibco, Waltham, MA, USA) for 6 or 16h, respectively, for activation or apoptosis assay. Next, exosomes (50 μL of the total fraction, CD45(+), or CD45(−) fraction) isolated from plasma of HNSCC patients were added and co-cultures were incubated for 16 or 6h, respectively, for activation or apoptosis assay. Only the CD45(+) fraction was bound on beads. Co-cultures containing no exosomes (50 μL PBS alone) or streptavidin beads with biotinylated CD45Ab served as negative controls. Apoptosis of CD8^+^ T cells and CD69 expression were measured by flow cytometry with a Gallios flow cytometer using an apoptosis assay kit (Annexin V-FITC Apoptosis Staining/Detection Kit, Abcam, Cambridge, UK) and CD69-FITC antibody (555530, BD Pharmingen, Franklin Lakes, NJ, USA), respectively. The protocol was previously described in detail [14].

#### 4.6.2. CD39 Induction in CD4+ T Cells by Patients’ Exosomes

Exosomes of HNSCC patients were co-incubated with healthy human CD4(+) T cells (10^5^/100 µL) in the presence of 20 μM of exogenous ATP (Sigma Life Science). Exosome samples used were derived from the total, CD45(+), or CD45(−) fraction. The CD4(+) T cells were isolated using a CD4+ T cell isolation kit (17952, Stemcell Technologies) according to manufacturer’s instruction. Control samples incubated with no exosomes (PBS only) and without ATP were used.

After 20h of incubation, percentages of CD4+CD39+ T cells were determined by flow cytometry using anti-CD4-FITC Ab (11-0049-42, eBioscience) and anti-CD39-PECy7 Ab (25-0399-42, eBioscience, San Diego, CA, USA). The protocol was previously described in detail [8,20,31].

#### 4.6.3. Evaluation of Adenosine Production by Patients’ Exosomes

Total, CD45(−), and CD45(+) exosomes were concentrated to 10 µg in 100 µL and incubated with 20µM ATP for 1h at 37 °C. Exosomes without ATP or ATP alone were used as controls. All samples were then centrifuged at 6000 *g* for 2 min at RT and boiled for 2 min at 95 °C. The samples were stored at −80 °C until further processing. 5′-AMP, adenosine, and their degradation products inosine, hypoxanthine, and xanthine were measured by mass spectrometry. Using liquid chromatography-tandem mass spectrometry, the selected reaction with ^13^C_10_-adenosine as an internal standard was measured. 1% acetic acid (mobile phase 1) and methanol (mobile phase 2) were used as mobile phases in differing elution gradients. The mass spectrometer was outfitted with a heated electrospray ionization source and operated in positive-ion mode. The method was previously described by our group [8,31].

### 4.7. Statistics

Statistical analysis was performed using Prism version 7 (Graphpad, San Diego, CA, USA). Scatter dot plots depict mean and standard error of mean (SEM) or standard deviation (SD), as stated in the figure legends. As a nonparametric test of unpaired values, the Mann-Whitney test was chosen. The *p*-value of <0.05 was used to evaluate the significance of the data.

## 5. Conclusions

Exosomes derived from plasma of UICC high stage HNSCC patients showed higher immune-suppressive effects than exosomes from plasma of UICC low stage patients. Furthermore, we show for the first time that effects of CD45(−) and CD45(+) exosomes differ substantially, allowing us to gain further insights into TEX- and non-TEX-mediated changes in the TME. Although CD45(+) exosomes have an immune-suppressive potential, the highest immune suppression is caused by TEX. Due to their origin and obviously distinguishing characteristics, CD45(−) TEX-enriched exosomes can be considered as an easily accessible liquid biomarker for cancer aggressiveness in HNSCC. Additionally, the CD45(+) hematopoietic exosomes can be used as a biomarker for immune suppression in the TME. 

## Figures and Tables

**Table 1:** Patient characteristics.

Characteristics.	Patients (*n* = 41)	
	*n*	%
Age (years)		
≤ 65	29	71
> 65	12	29
(range: 43–85)		
Gender		
Male	38	93
Female	3	7
Primary tumor site		
Oral cavity	14	34
Pharynx	21	52
Larynx	5	12
Squamous cell skin cancer (ear)	1	2
Tumor stage		
T1	13	32
T2	12	29
T3	12	29
T4	4	10
Nodal status		
N0	19	46
N+	22	54
Distant metastasis		
M0	41	100
UICC stage		
I/II	19	46
III/IV	22	54
Alcohol consumption		
Yes	18	44
No	22	54
Unknown	1	2
Tobacco consumption		
Yes	26	63
No	15	37

## Supplementary Materials

The following are available online at https://www.mdpi.com/2072-6694/12/7/1997/s1, Figure S1: Uncropped Western blots and densitometry results of Figure 1.
