# Supplementary material for: Immune Suppressive Effects of Plasma-Derived Exosome Populations in Head and Neck Cancer
Source: Cancers (Basel). 2020 Jul 21;12(7):1997. doi: 10.3390/cancers12071997 (PMC7409343; doi:10.3390/cancers12071997)

Supplementary Data

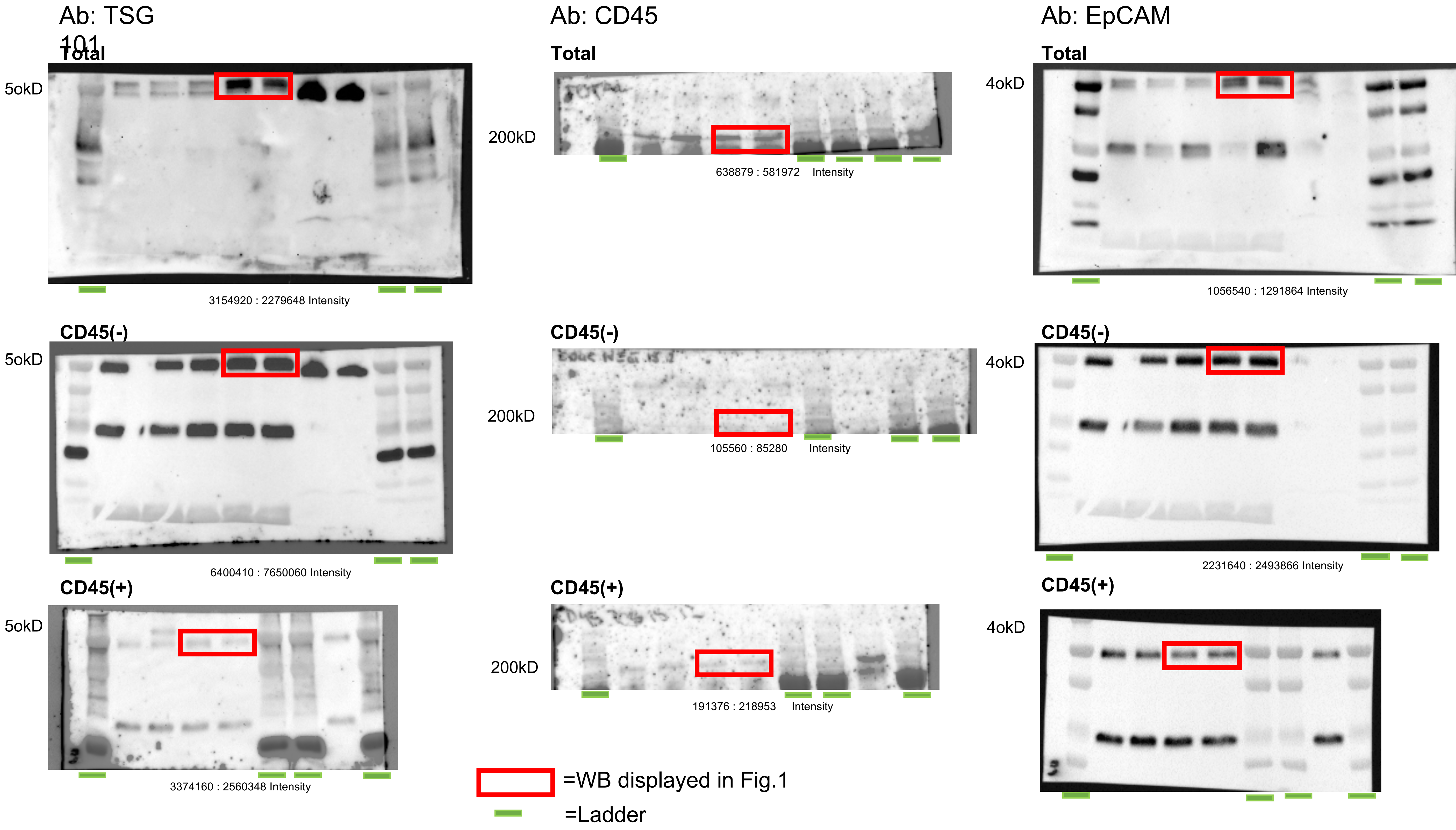

Supplement: Supplementary file 1 [file cancers-12-01997-s001.pdf]
